# Simple nested Bayesian hypothesis testing for meta-analysis, Cox, Poisson and logistic regression models

**DOI:** 10.1038/s41598-023-31838-8

**Published:** 2023-03-23

**Authors:** Klaus Rostgaard

**Affiliations:** 1 Danish Cancer Society Research Center, Copenhagen, Denmark; 2 Department of Epidemiology Research, Statens Serum Institut, Copenhagen, Denmark

**Keywords:** Medical research, Risk factors, Mathematics and computing

## Abstract

Many would probably be content to use Bayesian methodology for hypothesis testing, if it was easy, objective and with trustworthy assumptions. The Bayesian information criterion and some simple bounds on Bayes factor are closest to fit this bill, but with clear limitations. Here we develop an approximation of the so-called Bayes factor applicable in any bio-statistical settings where we have a d-dimensional parameter estimate of interest and the d x d dimensional (co-)variance of it. By design the approximation is monotone in the *p* value. It it thus a tool to transform *p* values into evidence (probabilities of the null and the alternative hypothesis, respectively). It is an improvement on the aforementioned techniques by being more flexible, intuitive and versatile but just as easy to calculate, requiring only statistics that will typically be available: e.g. a *p* value or test statistic and the dimension of the alternative hypothesis.

## Introduction

The majority of epidemiological studies are exercises in measurement; i.e., we try to estimate as accurately as we can some potential (possibly causal) association between an exposure and an outcome. Occasionally it is also of substantive interest to assess the evidence in favor of the null hypothesis, e.g. in terms of a probability of the null hypothesis. When assessed as probabilities this requires that the evidence in favor of the alternative(s) is assessed too. This is not possible in the traditional frequentist approach to statistical inference, as it is only based on the expectations that flows from assuming a particular null data generating mechanism/model. In the Bayesian approach it is possible to do it, but at the cost of having to specify some priors as input to the calculations. In the standard Bayesian paradigm these priors are supposed to model the beliefs of the investigator or client based on all relevant knowledge, not just studies or experiments similar to the one being analyzed. The subjectivism that flows from that is anathema to the standard scientific learning process, which is one reason why the standard frequentist approach is still dominant today. See Gilboa^1^ p. 40–48 for an excellent presentation of why you would want to act as a Bayesian in some situations and as a frequentist in other situations regarding the same substantive matters. However, it has often been demonstrated that the evidence for the alternative is weaker than usually recognized in the classical *p* value based scenario^2–4^. This would suggest that a Bayesian approach to model assessment would be preferable, if at all feasible.

In the following we shall use the terms model/data generating mechanism $M_0$ as synonomous to a null hypothesis $H_0$ and model/data generating mechanism $M_1$ as synonomous to the alternative hypothesis $H_a$, also denoted $H_1$. The full-blown Bayesian approach provides the probability of the null hypothesis after seeing the data $\varvec{D}$, $\textrm{pr}(M_0|\varvec{D})$ from the ratio $\textrm{pr}(M_1|\varvec{D})/\textrm{pr}(M_0|\varvec{D})$ which in turn is constructed from the product of the so-called Bayes factor and the prior odds $\textrm{pr}(M_1)/\textrm{pr}(M_0)$, see Eqs. (3) and (4). As Eq. (4) states the Bayes factor is the ratio of the probability of observing the data $\varvec{D}$ under the alternative model and the probability of observing the data under the null model, i.e. the Bayes factor is a ratio of predictive performance on the data $\varvec{D}$ of the data generating mechanisms/models $M_1$ and $M_0$. Hence in the Bayesian framework the Bayes factor is the sole modifier of prior beliefs about the model probabilities into posterior beliefs after having seen the data.

We will argue that it is possible to choose an objective informative “consensus” prior, essentially defined by requiring that the expression for Bayes factor in the case of a univariate interest parameter, generalizes in the natural way to the multivariate case thereby ensuring that the evidence in favor of the alternative is monotone in the likelihood-ratio and hence the *p* value. Unlike the situation for parameter estimation, Bayes factors depend critically on the priors over the interest parameter $\varvec{\theta }$: $p_1(\varvec{\theta })$ for $M_1$ and $p_0(\varvec{\theta })$ for $M_0$ (the latter is trivial), which therefore cannot just be made “uninformative” at no cost, see Kass & Raftery^5^.

We will argue that the ultimate (pre-data) prior odds ($\textrm{pr}(M_1)/\textrm{pr}(M_0)$) in an objective scientific setting should be set to 1. Readers may enter their own subjective (pre-data) prior odds into Eq. (3) and revise posterior inferences accordingly. Often this is all we ask for when we are discussing what our particular study adds (through Bayes factor) to the body of knowledge about the potential association between exposure $\varvec{X}$ and outcome $\varvec{Y}$.

We provide a simple, defendable, objective way of generating $p_1(\varvec{\theta })$ and the ensuing inferences, including the Bayes factor, applicable to situations where the data likelihood does not contain a dispersion parameter or its value can be assumed effectively known. Thus our methodology is immediately applicable in the many epidemiological studies where the interest parameters are estimated using e.g. logistic regression, Poisson regression or Cox regression.

For an accessible overview of Bayesian methodology for epidemiologists and contrasts to traditional statistics, see Wagenmakers et al.^6^.

The disposition of the paper is as follows. The next section develops and motivates the method. In the first subsection we introduce the setting and notation. The next subsection fully develops our consensus priors for the case of a univariate interest parameter including choosing the only free parameter $\lambda$ that expresses a balance of information content between prior and data. The next subsection swiftly generalizes this methodology to the general multivariate case. The following section compare our approach to existing approaches including the Bayesian Information Criterion (BIC). In the next section we elaborate our view on how to choose the pre-data prior odds, and discuss alternatives. The next section provides an epidemiological example of why we need this Bayesian approach (we believe in $H_0$) and what comes out of it, and illustrates considerations regarding alternative values of $\lambda$. We end the paper with a discussion of mainly how the inferences obtained from using our machinery differs from those obtained with traditional frequentist means.

## The method

### Setting and notation

We only consider interest parameters summarized in a parameter (vector) $\varvec{\theta }$ and assume large-sample asymptotics, i.e. everything is treated as multivariate normal $N_d(\cdot ,\cdot )$. Thus we use the same assumptions underlying the standard statistical software output of parameter estimates with associated standard errors, confidence limits, $\chi ^2$-based test statistics etc used when analyzing Cox, Poisson and logistic regression models. Stated differently, we at most assume known the maximum likelihood interest parameter estimates and their associated observed covariance-matrix (a submatrix of the inverted observed Fisher information matrix), as would be used as the input for a (multivariate) meta-analysis^7^. These statistics and various test statistics based upon them are the only data that you are always likely to be allowed to communicate in studies of humans. This is a first order approximation to a much more elaborate and accurate calculation of the Bayes factor that would only be possible to do for some-one with access to all original raw data. On the other hand this asymptotic approximation yields fully efficient parameter estimation under very reasonable assumptions^8^. The approach here is an easy addition on top of standard analysis output, and in the end allows us to retrospectively apply it to previous studies using only a few test statistics that should often be available to us (e.g. *p* values), in line with other model selection criteria like the Akaike Information Criterion (AIC), the BIC and various test-based bounds on the Bayes factor, as surveyed in Held & Ott^9^. The methodology developed here applies equally to the summary of a single study as a meta-analysis style summary of multiple studies.

The notation for the univariate case ($d = 1$), where all the relevant vectors and matrices can be treated as scalars (numbers) is as follows:

- Data $$\varvec{D}$$: $$L(\varvec{D},\theta ) = L_0 \exp (-\frac{1}{2}(\theta -\hat{\theta }) V^{-1}(\theta -\hat{\theta }))$$,
- Prior $M_0$: $p_0(\theta ) = \delta _{0}$ (all probability mass in the point 0),
- Prior $$M_1$$: $$p_1(\theta ) = N(\theta _1,W)$$.

Let $$K\equiv V^{-1}$$ and $$P\equiv W^{-1}$$.

We have:

1 $$\begin{aligned} \frac{\textrm{pr}(M_1|\varvec{D})}{\textrm{pr}(M_0|\varvec{D})} = & {} BF_{10} \times \frac{\textrm{pr}(M_1)}{\textrm{pr}(M_0)} \end{aligned}$$

2 $$\begin{aligned} BF_{10}\equiv & {} \frac{\textrm{pr}(\varvec{D}|M_1)}{\textrm{pr}(\varvec{D}|M_0)} = \frac{\int L(\varvec{D},\theta )p_1(\theta )\ d\theta }{\int L(\varvec{D},\theta )p_0(\theta )\ d\theta } \end{aligned}$$

In order to calculate $BF_{10}$ we have to choose $\theta _1$ and *W*, the mean and covariance respectively for the a priori distribution of $\theta$.

The notation for the general case is as follows:

- Data $$\varvec{D}$$: $$L(\varvec{D},\varvec{\theta }) = L_0 \exp (-\frac{1}{2}(\varvec{\theta }-\hat{\varvec{\theta }})^t \varvec{V}^{-1}(\varvec{\theta }-\hat{\varvec{\theta }}))$$,
- Prior $M_0$: $p_0(\varvec{\theta }) = \delta _{\varvec{0}}$ (all probability mass in the point $\varvec{0}$),
- Prior $$M_1$$: $$p_1(\varvec{\theta }) = N_d(\varvec{\theta }_1,\varvec{W})$$.

Let $$\varvec{K}\equiv \varvec{V}^{-1}$$ and $$\varvec{P}\equiv \varvec{W}^{-1}$$.

We have:

3 $$\begin{aligned} \frac{\textrm{pr}(M_1|\varvec{D})}{\textrm{pr}(M_0|\varvec{D})} = BF_{10} \times \frac{\textrm{pr}(M_1)}{\textrm{pr}(M_0)} \end{aligned}$$

4 $$\begin{aligned} BF_{10}\equiv \frac{\textrm{pr}(\varvec{D}|M_1)}{\textrm{pr}(\varvec{D}|M_0)} = \frac{\int L(\varvec{D},\varvec{\theta })p_1(\varvec{\theta })\ d\varvec{\theta }}{\int L(\varvec{D},\varvec{\theta })p_0(\varvec{\theta })\ d\varvec{\theta }} \end{aligned}$$

In order to calculate $BF_{10}$ we have to choose $\varvec{\theta }_1$ and $\varvec{W}$ of dimension *d* and $d\times d$, respectively. $\varvec{\theta }$ and other vectors are column vectors. $\varvec{\theta }^t$ denotes $\varvec{\theta }$ transposed.

Note that much literature on Bayes factors, including Held & Ott^9^ and Wagenmakers et al.^6^, gives formulas for and bounds on $BF_{01} = BF_{10}^{-1}$, while we prefer to use $BF_{10}$ to highlight similarities to usual penalized likelihood methods.

### An asymptotic Bayes factor for a univariate hypothesis ($d = 1$)

Taking as starting point the typical epidemiological research question “Does $\varvec{X}$ affect the risk of $\varvec{Y}$ in any way?” and it’s classical statistical formulation as $H_0: \theta = 0$ versus an alternative of no such constraint on $\theta$ clearly suggests that the prior $p_1(\theta )$ should be centered at $\theta _1 = 0$. We may have a hunch about the direction of an effect, but noting how rarely anyone dares to consider only one-sided hypotheses etc it seems irrelevant to consider other values than 0 as the center of the prior. Or stated differently: If we were very certain about where $\theta _1\ne 0$ should be, we probably would not need to assess $\textrm{pr}(M_0)$ or the Bayes factor in the first place. Other desiderata that we may consider, e.g. that $\theta _1$ should be simple, unique, self-evident, biased towards $H_0$/$M_0$ etc, would point in the same direction. 0 simply seems to be the only point that could possibly fulfill most desiderata.

Assume the above expressions for the priors and likelihood and $\theta _1 = 0$. For reasons to become apparent let $P = \lambda K$, $\psi \equiv \lambda /(1+\lambda )$ and $LR = \exp (\frac{1}{2}\hat{\theta } K \hat{\theta })$. Then

5 $$\begin{aligned} BF_{10} = & {} LR \psi ^{1/2} \exp \left( -\frac{1}{2}[\psi \hat{\theta } K\hat{\theta }]\right) \end{aligned}$$

6 $$\begin{aligned} = & {} \psi ^{1/2}LR^{1-\psi } \end{aligned}$$

In deviance form (6) is $\log BF_{10} = \frac{1}{2} \log {\psi } +\frac{1-\psi }{2}\chi ^2$ where $\chi ^2$ is the difference in deviance between models 0 and 1. See Supplementary Eqs. E1 & E2 for derivations.

$\lambda$ is a ratio between the information in the prior and the data formalized as $P = \lambda K$. So any formula for calculating $\lambda$ should reflect this, e.g. $\lambda ^{-1}\propto K$, and $\lambda \downarrow 0$ as more data are gathered. If we choose $\lambda$ large the alternative $\theta$ will be shrunk very much towards 0 and $M_1$ will look very similar to $M_0$ and Bayes factor will by necessity be close to 1, i.e. we essentially learn nothing from our data, the inference is what we put into the model in the form of the prior. If on the other hand we make $\lambda$ too small we are always going to prefer $M_0$, due to having spread out the probability mass too thinly and hence placed very little in the vicinity of $\hat{\theta }$. This never-vanishing importance of the choice of the prior when testing hypotheses stands in glaring contrast to the situation where we estimate parameters. Here the choice of prior is usually not very important, because as the amount of data increases the posterior distribution will converge to the same limiting distribution^5,6^.

Bayes factor is maximized by $\psi = 1/ \hat{\theta } K \hat{\theta } \cong \lambda$ for $\lambda \ll 1$ so $\lambda =1/\hat{\theta } K \hat{\theta }$ is not too small and shrinks at the right pace as more data are gathered and effectively maximizes the evidence in favor of the alternative. However, this $\lambda$ may be too large. We may actually believe in the null as an appropriate approximation of the truth and want Bayes factor to favor the null ($BF_{10}<1$) and more so the smaller $\theta K \theta$ is below some value. We may obtain this by introducing an upper limit to how large $\lambda$ may be. Consider $\log BF_{10} = \frac{1}{2}\log \psi + \frac{1-\psi }{2}\chi ^2$. $BF_{10} = 1$ when $\log \psi = (\psi -1)\chi ^2$. This only has a solution besides $\psi = 1$ when $\chi ^2 > 1$. E.g. the solution to the Equation with $\chi ^2 = 2$, corresponding to $\lambda _{max}\approx 0.255$ yields preferences similar to applying the Akaike information criterion (AIC): when the decrease in deviance per dimension is larger than 2 we prefer the alternative, complicated model, when the decrease in deviance per dimension is smaller than 2 we prefer the simpler model ($H_0$). Likewise if we choose $\chi ^2 = 3.92$ as our “watershed”, corresponding to the usual $p = 0.05$ accept/reject dichotomy for a one-dimensional hypothesis, this corresponds to $\lambda _{max}\approx 0.022$.

**Figure 1:**
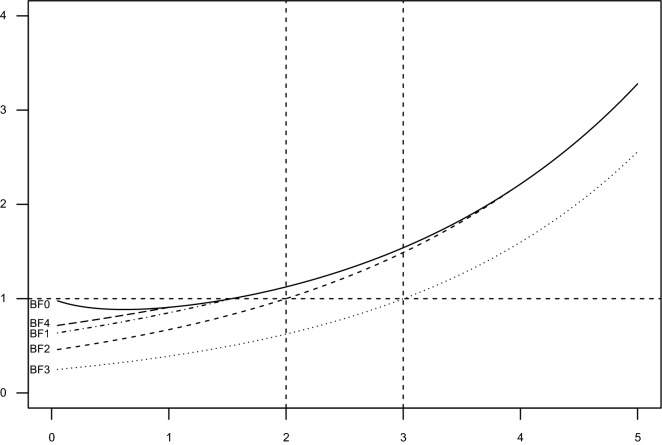
Bayes factor as a function of $\chi ^2$ and $\lambda$. BF0: $\lambda = 1/\chi ^2$, BF1: $\lambda = 1$, BF2: $\lambda = 0.255$, BF3: $\lambda = 0.063$, BF4: $\lambda = 0.65$.

Examining the proposal of employing a $\lambda _{max}$ more closely reveals features that may guide the choice of $\lambda _{max}$. When $\lambda = 1/\chi ^2$ throughout Bayes factor is not completely monotonely decreasing in $\chi ^2$ (Fig. 1), yielding an argument for introducing a $\lambda _{max}$ small enough to ensure monotonicity. Furthermore if we require that Bayes factor becomes 1 at some prespecified watershed then we have to require $\lambda _{max}<1/1.54 = 0.65$ corresponding to $\chi ^2>1.54$. Thus there is actually little leeway to choose a sensible $\lambda _{max}>0.255$ and we would therefore argue against that. It may however make good sense in specific situations to choose $\lambda _{max}<0.255$.

We therefore propose as default

7 $$\begin{aligned} \lambda = \min (1/\hat{\theta } K \hat{\theta },\lambda _{max}) \end{aligned}$$

or more generally

8 $$\begin{aligned} \lambda = \min (1/\Delta DEV_{01},\lambda _{max}) \end{aligned}$$

where $\Delta DEV_{01}$ is the change in deviance between models 0 and 1, and $\lambda _{max}\le 0.255$. The corresponding Bayes factor is now a continuous monotonely increasing function of $\Delta DEV_{01}$. For $\Delta DEV_{01}<1/\lambda _{max}$ Bayes factor is a simple exponential function, reaching its minimum value of $\psi _{max}^{1/2}$ for $\Delta DEV_{01} = 0$.

In some studies we would be more lenient towards formally statistically significant results, either because we would suspect various biases that we could not mitigate or because the effect sizes we detect as statistically significant would not amount to a practically or clinically meaningful difference. It is the same kind of logic that persuades professional surveyers not to make their studies as large as logistically possible because random fluctuations are soon swamped by inevitable biases as sources of error^10^. So we could augment $\lambda _{max}$ according to such a “practically null” criterion. Then the watershed $\chi ^2$ would be on the form $T^2/V$ where *V* is the variance of the parameter, e.g. estimated from the width of the relevant confidence limits and |*T*| is the largest effect size we would tolerate as being in favor of the null hypothesis.

$\lambda$ being a ratio of information in the prior and the data suggests choosing $\lambda = \nu /\mu$ where $\nu$ and $\mu$ are counts of some information carrying unit as yet another way of specifying $\lambda$ and the watershed in a way that is objective, transparent and transportable. In survival analysis (Cox regression, Poisson regression) the growth of statistical information as the sample grows is reflected more accurately in the number of events observed (= the number of uncensored survival times) than the number of observational units^11,12^. This suggests that $\mu$ be the number of observed events in the data in survival analysis, perhaps just the number of observed events among the exposed, if exposure is rare. $\nu$ would then be the equivalent postulated information content in the prior—it would seem equivalent to empirical data containing $\nu$ events of the type counted by $\mu$.

Suppose that $\hat{\theta }\rightarrow \tilde{\theta } \ne 0$ as more data are gathered. Then $W = \lambda ^{-1}V=(\hat{\theta } V^{-1} \hat{\theta })V \rightarrow \tilde{\theta }^{2}$, i.e. the limiting prior variance is then well-defined and constant, thus mimicking having chosen a priori and subjectively a fixed $W=\tilde{\theta }^2$. Furthermore the region around 0 where we prefer $H_0$ is on the form $\{\theta : \theta K \theta<c \Leftrightarrow |\theta | < \sqrt{cV}\}$, where *V* is halved every time we double the number of observations (*n*) or other information carrying units. Thus the size of this region will be shrinking at the pace of $\sqrt{n}$. Through the device of requiring $\lambda \le V/T^2$ to accomodate a practical null result this shrinkage can be halted, to make this region asymptotically fixed. If $\tilde{\theta }$ is within it we will asymptotically prefer $H_0$, if it is outside that region we will asymptotically end up preferring $H_1$ and the evidence in favor of $H_1$ measured by the Bayes factor will become infinite.

Our approach has been to constrain a hypothetical subjectively specified prior in ways that would make it objective. Evidently we have succeeded in generating a recipe for such a prior that asymptotically behaves as if fixed a priori and subjectively. Conversely, one may ask if this prior is likely also to be a consensus subjective prior in the sense of representing subjective beliefs in the scientific community on the subject matter to an acceptable degree? The traditional subjective prior $N(\theta _1,W)$ allows us to specify beliefs about $\theta$ as a location ($\theta _1$) and a degree of uncertainty about this location (*W*). Our new prior has introduced the constraint $\textrm{E}\theta =\theta _1=0$ and thus we are forced to express our prior beliefs about $\theta$ by specifying beliefs only about $\textrm{E}\theta ^2=\textrm{Var}_S(\theta )+(\textrm{E}_S \theta )^2=\lambda ^{-1}V$, where we have used *S* to designate subjective quantities. This will only potentially be very different from employing the prior $N(\theta _1,W)$ when $|\theta _1/W|$ is large. However, if we were so sure about where $\theta$ was located (without having looked at the data!), it would seem more appropriate to make the comparison between $H_0$ and $H_1$ a comparison between two simple hypotheses, i.e. $H_1$ would be the hypothesis that $\theta =\theta _1$. We have elaborated on bounds on how large $\lambda$ should be allowed to be. Imagine a subjectively specified $\lambda _S \ll \min (1/\Delta DEV_{01},\lambda _{max})$. This would almost surely be a consequence of believing in numerically larger effect sizes than what turned out to be the case, and as such signifying beliefs in $H_1$, i.e. that $\theta$ was far from 0. A person having such beliefs should be happy to be “corrected” in the direction of more evidence for $H_1$ by our consensus prior. Altogether, we believe that many would-be practitioners of subjective Bayesianism in science would be relieved and happy to employ our admittedly flexible consensus prior for scientific nested model comparison.

Classical Bayesian inference using Bayes factors may suffer from Lindley’s paradox, which has caused some to suggest abandoning Bayes factors altogether for nested hypothesis testing^13^. In our setup the paradox corresponds to imagining a sequence of test statistics ($\chi ^2$s) that are constant, but corresponding to monotonically decreasing *V*s as more data are gathered^13^. In that case the Bayes factor will at some point start to favor the null hypothesis over the alternative to an arbitrary degree, despite the test statistic being fixed at some level that would usually by the scientist be considered strongly in favor of $H_1$. If we use $\lambda =\nu /\mu$, letting $\mu \rightarrow \infty$ and hence $\lambda \rightarrow 0$, and fix the test statistic and hence the $LR=LR_0$ we have $BF_{10}\approx \sqrt{\lambda }LR_0\rightarrow 0$, indeed exhibiting Lindley’s paradox. However, the standard data-driven version of Bayes factor proposed here does not suffer from the paradox: the only $\chi ^2$s that will make us favor $H_0$ are those that we deliberately through our choice of a watershed $\chi ^2$ have designated as being in favor of $H_0$.

### A general asymptotic Bayes factor ($d\ge 1$)

When generalizing our Bayes factor from one dimension to multiple dimensions it would seem natural to have a formula and priors that do so too. This is indeed possible. Let $\varvec{\theta }_1=\varvec{0}$ and $\varvec{P}=\lambda \varvec{K}$ and let $\psi \equiv \lambda /(1+\lambda )$ and $LR=\exp (\frac{1}{2}\hat{\varvec{\theta }}^t \varvec{K}\hat{\varvec{\theta }})$ and assume the above expressions for the priors and likelihood. Then

9 $$\begin{aligned} BF_{10}= & {} LR \psi ^{d/2} \exp \left( -\frac{1}{2}[\psi \hat{\varvec{\theta }}^t \varvec{K}\hat{\varvec{\theta }}]\right) \end{aligned}$$

10 $$\begin{aligned}= & {} \psi ^{d/2}LR^{1-\psi } \end{aligned}$$

In deviance form (10) is $\log BF_{10}=\frac{d}{2} \log {\psi } +\frac{1-\psi }{2}\chi ^2$ where $\chi ^2$ is the difference in deviance between models 0 and 1. See Supplementary Eqs. E3–E5 for derivations. Obviously monotonicity in *LR* and hence the *p* value has been maintained.

$\varvec{P}$ may also be essentially obtained from requiring monotonicity of the the Bayes factor in the *p* value, rather than for esthetic and computational reasons, as elaborated below.

The precision matrix of the study $\varvec{K}=\varvec{V}^{-1}$ is often called the information matrix: it tells us what our study is most informative about; which parameters can be estimated with the biggest precision. Thus the standard Wald test statistic $\hat{\varvec{\theta }}^t \varvec{K} \hat{\varvec{\theta }}$ essentially collects evidence against the null, penalizing deviations from the null of a given size harder in directions where the sample/study is informative than in directions where it is less informative. To be more specific: any covariance matrix, and it’s inverse, has a representation as a diagonal matrix, i.e. $\varvec{K}=\varvec{O}^t \varvec{\Lambda } \varvec{O}$ where $\varvec{O}$ is a rotation matrix and $\varvec{\Lambda }=\textrm{diag}(\lambda _1,\ldots ,\lambda _d)$ with $\lambda _1 \ge \ldots \ge \lambda _d$. Thus the Wald test statistic $\hat{\varvec{\theta }}^t \varvec{K} \hat{\varvec{\theta }}$ has a representation in another basis (specified by $\varvec{O}$) on the form $\sum _{i=1}^{d} w^2_i \lambda _i$. Noting that most sources of information about $\varvec{\theta }$ are likely to resemble our study and the correlations between components of $\varvec{\theta }$ to be roughly similar choosing the prior precision $\varvec{P}\propto \varvec{K}$ therefore seems an obvious idea. Also if we view the Bayes factor as an extension of the traditional significance test we may insist that the Bayes factor in favor of the alternative should increase monotonically as the *p* value decreases. In our set-up such a constraint can be honored, but it requires that $\varvec{P}$ is diagonal in the same basis as $\varvec{K}$ and further puts restrictions on the ranks of the eigenvalues of $\varvec{P}$. If we furthermore require that any scale copy of $\varvec{P}$ should obey the *p* value monotonicity constraint, the eigenvalues of $\varvec{P}$ has to obey the same ranking as the eigenvalues of $\varvec{K}$, see Supplementary Equation E6 for derivations. The only practically viable option for obtaining this is to have $\varvec{P}=c_1\varvec{I}+c_2\varvec{K}$, with $c_1, c_2 \ge 0$, noting that $c_1 \varvec{I}$ has the same representation in all bases. The mean of the posterior of $\varvec{\theta }$ under $M_1$ will be $\varvec{m}=(\varvec{K}+\varvec{P})^{-1}\varvec{K}\hat{\varvec{\theta }}$ which will be exactly in the direction of $\hat{\varvec{\theta }}$ only when $\varvec{P}\propto \varvec{K}$, or stated differently: $\varvec{m}$ can only be interpreted as merely shrinking $\hat{\varvec{\theta }}$ towards $\varvec{0}$ in case $\varvec{P}\propto \varvec{K}$, which is trivially fulfilled when $d=1$.

Further in favor of using $\varvec{P}\propto \varvec{K}$ we note that the information matrix $\varvec{K}$ for Poisson and logistic regression models is derived from a larger information matrix on the form (in standard notation) $\varvec{X}^t\varvec{W}\varvec{X}$ where $\varvec{X}$ is an a priori known design matrix and $\varvec{W}$ is a diagonal matrix of weights of each observation in the data set. These weights will in general depend on $\varvec{\theta }$ but even then $\varvec{K}$ will typically deviate little from $\varvec{K}(\varvec{\theta }_1=\varvec{0})$, i.e. $\varvec{K}$ calculated based on the weights corresponding to the null hypothesis, especially when the effect sizes are small. The expression for the information matrix in Cox regression is more complicated than for Poisson and logistic regression, but the same argument applies; that the difference between $\varvec{K}$ and $\varvec{K}(\varvec{\theta }_1=\varvec{0})$ is likely to be small, vindicating the use of $\varvec{P}\propto \varvec{K}$.

So $\varvec{P}=\lambda \varvec{K}$ is not only very convenient, yielding simple formulas; it is also deeply meaningful as the best suggestion absent other prior knowledge about what $\varvec{P}$ should be. For other examples of approaches where the specification of the details of the prior distribution regarding correlation structure etc is based on the data see Chen & Ibrahim^14^ and Bedrick et al.^15^.

We have used Eqs. 9 and 10 and derivatives thereof interchangeably. The former is what comes out of our modeling, while the latter is an interpretation of it that provides the more general, robust and accurate way to calculate Bayes factor according to our ideas. Use of Eq. (10) and hence deviance ($\chi ^2$) firstly guarantees that our inference is indeed monotone in the LR and hence in the *p* value, secondly is in better correspondence with the Savage–Dickey density ratio theorem, which states that Bayes factor quite generally can be calculated by dividing the prior in $\varvec{0}$ by the posterior in $\varvec{0}$ both under $H_1$, i.e. $BF_{10}=p_1(\varvec{0})/p_1(\varvec{0}|\varvec{D})$^6^. The Savage–Dickey density ratio theorem also provides an argument why the calculation of Bayes factor should be insensitive to specification of priors and likelihoods for nuisance parameters^6^.

In Supplementary Equation E7 we provide further heuristic arguments why a universal Bayes factor should look like Eq. (10), using the Savage–Dickey density ratio theorem^6^.

If $\lambda$ is not chosen prior to seeing $\varvec{\theta }$, then it must be a function of $\varvec{\theta }^t \varvec{K} \varvec{\theta }$ to ensure monotonicity in the *p* value; i.e. all $\varvec{\theta }$ for which $\varvec{\theta }^t \varvec{K} \varvec{\theta }=c$ should lead to the same $\lambda$ to obtain the same inference.

Bayes factor is maximized by $\psi =d/ \hat{\varvec{\theta }}^t \varvec{K} \hat{\varvec{\theta }} \cong \lambda$ for $\lambda \ll 1$ so $\lambda =d/\hat{\varvec{\theta }}^t \varvec{K} \hat{\varvec{\theta }}$ is not too small and shrinks at the right pace as more data are gathered and effectively maximizes the evidence in favor of the alternative.

So we immediately end in the natural generalization of our proposed $\lambda$ estimator for $d=1$:

11 $$\begin{aligned} \lambda =\min (d/\hat{\varvec{\theta }}^t \varvec{K} \hat{\varvec{\theta }},\lambda _{max}) \end{aligned}$$

or more generally

12 $$\begin{aligned} \lambda =\min (d/\Delta DEV_{01},\lambda _{max}) \end{aligned}$$

where $\Delta DEV_{01}$ is the change in deviance between models 0 and 1, and $\lambda _{max}\le 0.255$. The corresponding Bayes factor is now a continuous monotonely increasing function of $\Delta DEV_{01}$. For $\Delta DEV_{01}<d/\lambda _{max}$ Bayes factor is a simple exponential function, reaching its minimum value of $\psi _{max}^{d/2}$ for $\Delta DEV_{01}=0$.

In the general case we may also want to use a “practically null” criterion to put an upper bound on $\lambda$. For ease of interpretation and communication we suggest that such a criterion should typically be based on a one-dimensional margin of the interest parameter.

## Connections to other theory

Most approaches to “objective” statistical inference have more or less equated “objective” with using minimally informative or even improper priors, including the fiducial approach by Fisher and in the same spirit, the *p* value function by Fraser^16–18^. Inherently, this in many cases clearly favors the null hypothesis in nested hypothesis testing. Our approach to “objective” statistical inference here is the complete opposite. Our starting point is that modern “subjective” Bayesian statistics in the tradition of Savage and others logically and otherwise works fine^1,19^, the only real defect in the scientific context being that it may be accused of being “subjective” in its choice of priors. So our project here has been to examine which sensible constraints on the priors could turn this methodology into an objective methodology in the spirit and self-image of hard science. As argued earlier we believe we have managed not just to provide a data-driven objective prior, but also at the same time a likely consensus subjective prior.

In its classical form the Minimum Description Length (MDL) principle used for model selection to a first approximation corresponds to using the AIC^20^ and as such is likely to yield inferences very similar to the inferences we would obtain from the default version of our Bayes factor. Later developments of the MDL principle have had a less Bayesian flavor^21,22^. However, the MDL principle and similarly looking penalized likelihood methods^22,23^ do not seem to match our consensus prior regarding flexibility, ease of calculation and ease of interpretation.

In essence BIC is obtained from our approach by insisting on $\lambda =\nu /\mu$ where $\nu$ and $\mu$ are counts of some information carrying unit. In the BIC $\nu =1$, corresponding to a prior with the information content of a single average observation (or whatever unit we are counting) and as such as little information in the prior as empirically conceivable.

A frequently proposed upper bound on Bayes factor is $1/(-e p \log (p))$^24^. This and other bounds on Bayes factor are surveyed in Held & Ott^9^. However, neither of these bounds nor the BIC admits the flexibility and realism of our approximation of Bayes factor. E.g. the BIC has a clear tendency to favor $H_0$ and in the opposite direction the aforementioned bound on Bayes factor always yields $BF_{10} \ge 1$ corresponding to letting $\psi \rightarrow 1$ and thus using improbably precise priors, rendering the data irrelevant for inference.

We also note that $BF_{10}=\psi ^{d/2}LR^{1-\psi }$ (Eq. 10) also appears as an approximate Bayes factor in work on the fractional Bayes approach (section 2 in O’Hagan^25^), suggesting a wider applicability of Eq. (10) than stated here. We don’t find this surprising since we also learn the prior from the data, although we have argued that this is merely for convenience; often the result should be very close to what we could learn from fitting $M_0$. However, we do part company with O’Hagan in our recommendations regarding $\psi$ (section 6 in O’Hagan^25^).

In Supplementary Equation E4 we have adapted our machinery for use in classical subjective Bayesian inference with a prior on the form $N_d(\varvec{\theta }_1,\lambda ^{-1}\varvec{V})$, thus solving an escalating logistic problem of eliciting/specifying very many parameters of the prior. Specifying $d+1$ meaningful parameters should certainly be doable.

Much of what we have developed here is foreshadowed by several decades, at least in the univariate case, by work from the inventor of the Bayes factor, Sir Harold Jeffreys^26^. Among other things Jeffreys developed approximate expressions for the Bayes factor very similar to (5) and (6), i.e. in its simplest form in our notation $BF_{10}\approx \sqrt{\lambda }LR$ thus realizing that the Bayes factor roughly is the product of a term that is a function of the *p* value (*LR*) and a term that depends on the square root of the information usually proportional to the number of observations *n* ($\sqrt{\lambda }$)^26^. He also realized that large and middle *p* values represents evidence in favor of $H_0$, not just absence of evidence for $H_1$^26^. Thus the novelty of the present contribution only lies in generalizing and specializing such type of approximative expression for Bayes factor to a broad class of regression models that are completely dominant in e.g. epidemiological research, providing arguments for choosing and scaling (through $\lambda$) the priors to be objective and possibly quite informative at the same time.

## Odds prior to data—and final inference

Before discussing pre-data prior odds of $M_0$ and $M_1$ we need to understand what the hypotheses really mean. If we were in a position to collect as much data as we would want we would probably in all but the rarest cases be able to identify an effect size different from $\varvec{0}$. So the meaning of $M_0$ is not really that we believe it to be absolutely true, but rather that we believe it to be so small as to be predictively indistinguishable from $\varvec{0}$ on potentially available data. Bayes factor in our context is a simplifying device. It collects evidence in favor of each hypothesis and therefore the null hypothesis of a simple model may not need to be abandoned until the evidence in favor of the alternative that you consider likely is much larger. As such a non-vanishing $\textrm{pr}(M_0|\varvec{D})$ is a license to ignore the true non-null effect size that we haven’t been able to pinpoint with sufficient precision. Just as a model is a simplification of reality, the null model is a simplification of an extended model. $H_0$ may be a hypothesis we wish to entertain, for convenience or simplicity, or something we wish to refute in order to demonstrate that some exposure affects the probability of some output, e.g. that some treatment is better than another treatment.

In epidemiology we are only likely to identify very small effect sizes with certainty when both the outcome and the exposure is very common, say when studying 30-day mortality following blood transfusion. Then even a tiny apparent relative difference in probability of the outcome by blood product characteristic, would, if true and causal translate into an actionable possibility of avoiding x adverse events per year. Non-trivial decision making is best done using decision theory. But if our interest lies in using Bayes factor as a simplifying device unless overwhelmed by evidence for the alternative we may do so by a slight change in the meaning of $H_0$ and $H_a$ to something closer to our implicit interpretation of $H_0$ and $H_a$ also in the situation with very abundant data by instead considering the posterior probability of $\varvec{\theta }$ being $\varvec{0}$ or practically $\varvec{0}$ ($\varvec{\theta } \in \varvec{R}_{\varepsilon }$), i.e. $\textrm{pr}(M_0|\varvec{D})+\textrm{pr}(\varvec{\theta }\in \varvec{R}_{\varepsilon }|M_1,\varvec{D})\textrm{pr}( M_1|\varvec{D})$. This is in the spirit of “modernizations” of the traditional significance test as advocated in Goodman et al.^27^ and Blume et al.^28^.

In order to obtain proper posterior odds and probabilities of hypotheses you need to asses (pre-data) prior odds of the models/hypotheses. In analogy with our choice of $\varvec{\theta }_1=\varvec{0}$ for the consensus parameter prior we consider pre-data prior model odds $\textrm{pr}(M_1)/\textrm{pr}(M_0)=1$ as the best possible practical universal consensus pre-data prior odds. Setting the prior odds equal to 1 corresponds to evaluating the posterior odds at the boundary between your a priori position, and the position of your adversary (who favors the opposite hypothesis) where the odds are as far in favor of your adversary’s point of view as you can accommodate. Further it could be argued that in keeping with the role of models as simplifying devices and Occam’s razor and the special role assigned to $H_0$ in science we should always have $\textrm{pr}(M_1)/\textrm{pr}(M_0)\le 1$. And on the other hand if we see the point of the test to be to possibly falsify/reject $H_0$ we should have $\textrm{pr}(M_1)/\textrm{pr}(M_0)\ge 1$. For an opposing view in favor of assessing/discussing the true pre-data prior odds in epidemiological studies, see Goodman et al.^29^.

There are other methods for determining pre-data prior odds, but they do not seem particularly reliable and objective in our view^30–33^. Anyway, the reader of your results can multiply their own pre-data prior odds with your objective Bayes factor to obtain their subjective posterior odds and probabilities.

Finally, we could avoid specifying pre-data prior odds of hypotheses altogether if we instead asked what is the expected posterior loss if we act as if some simplifying or interesting assumption ($H_0$) is true, measured in a big estimated model ($M_1$) we believe in^30^. But this of course requires an elaborate $M_1$ model and that you can obtain consensus with your readers/clients about what loss function to use.

## A practical example

We will illustrate the use of our methodology in an example concerning an eight-dimensional interest parameter, where we believe the null hypothesis to be a good approximation of the truth. We will show the simple calculations involved in assessing Bayes factor only based on statistics published in an epidemiological paper^34^.

Most people become infected by Epstein-Barr virus (EBV); once infected the virus persists in the host. In the western world primary EBV infection occurs mostly in infancy (0–3 years) and in teenage years. Occasionally primary EBV infection is accompanied by infectious mononucleosis; this happens rarely in infancy, but commonly in teenage-years and later. EBV is mostly transmitted through saliva and is not very contagious. Having siblings reduce the risk of infectious mononucleosis since each sibling may infect you with EBV in infancy, thereby pre-empting primary EBV infection in teenage-years with its associated larger risk of infectious mononucleosis. The protection against infectious mononucleosis obtained from each sibling varies widely by age difference; the smaller the difference in age, the more protection and with younger siblings being more protective than older siblings with the same absolute age difference to the followed-up person. This has been modeled in multiplicative (Poisson or Cox regression) models with time-varying counts of siblings in each of eight disjoint categories of age-difference as predictors^34^.

Infectious mononucleosis is a well-known risk factor for multiple sclerosis with hazard ratios (HRs) consistently in the range 2–3. Whether it is the infectious mononucleosis (an exaggerated immune reaction) per se, or infectious mononucleosis as a marker of so-called delayed EBV infection that is the culprit is unclear. But based on other evidence the latter seems most likely. If the latter was the case one should expect the HRs of multiple sclerosis as a function of sibship constellation to be the same as the HRs for infectious mononucleosis as a function of sibship constellation, as modeled by the aforementioned eight-dimensional predictor. This would then be our $H_0$. This was examined in a population-based study of persons born in Denmark since 1971 in a stratified Cox regression model with hospital contacts for multiple sclerosis and infectious mononucleosis, respectively as outcomes^34^. In this joint model the interest parameters are $\varvec{\theta }_{IM}=\varvec{\theta }$ and $\varvec{\theta }_{MS}=\varvec{\theta }+\varvec{\Delta \theta }$ with $H_0: \varvec{\Delta \theta }=\varvec{0}$. The details of the modeling are un-important here, it suffices to know that the hypothesis of common sibling parameter estimates for the two outcomes was examined using a likelihood-ratio test^34^.

It is obvious from the paper^34^ that the alternative hypothesis is 8-dimensional ($d=8$). And we are informed that the *p* value is 0.19, which with this *d* corresponds to a deviance $\chi ^2=11.21$. We thus obtain $\lambda =\min (8/11.21,0.255)=0.255$ and hence $\psi =\lambda /(1+\lambda )=0.255/1.255=0.203$. Plugging into $\log BF_{10}=\frac{d}{2} \log {\psi } +\frac{1-\psi }{2}\chi ^2$ yields $BF_{10}=\exp (-1.908)=0.1484$ and hence [under the assumption of uniform prior odds $\textrm{pr}(H_1)/ \textrm{pr}(H_0)=1$] we obtain the posterior probabilities $\textrm{pr}(H_1|\varvec{D})=0.129$ and $\textrm{pr}(H_0|\varvec{D})=0.871$. We consider these calculations uncontroversial and therefore perfectly adequate for the situation, suggesting that $H_0$ is indeed likely to be true. In this example the AIC actually puts a bound on $\lambda$. If we hadn’t used this bound we would instead have ended up in $BF_{10}=0.7923$ and thus $\textrm{pr}(H_1|\varvec{D})=0.442$ and hence $\textrm{pr}(H_0|\varvec{D})=0.558$  i.e. a result much closer to equiprobability of the two hypotheses as expected.

In the following we will examine various considerations that could potentially suggest a lower $\lambda$ than the one based on the AIC.

As an example of accomodating a practical null result let us consider the parameter for the effect of each additional 0–2 years younger sibling. This parameter has the largest effect size for the infectious mononucleosis outcome $|\log (HR)|=-\log (0.80)=0.223$. If we take *T* to be 20% of this effect size, to accomodate for instance that not all infectious mononucleosis is due to EBV we obtain $T=0.2\times 0.223=0.045$. The relevant variance estimate is obtained from $\sqrt{V}=(\log (1.19)-\log (0.89))/3.92=0.074$ where the confidence limits 0.89 and 1.19 belong to the estimate of the HR between the common HR for the two outcomes (=HR for infectious mononucleosis) and the HR for multiple sclerosis per sibling 0–2 years younger. The resulting $\chi ^2=0.045^2/0.074^2 = 0.36 \ll 2$ is useless. To obtain a useful $\chi ^2$ would require a much larger study (lower *V*) or that we were much more lenient in our choice of effect sizes favoring $H_0$ (larger *T*) or both.

Considering the one-dimensional case the AIC ($\chi ^2=2$) corresponds to using a significance level of $\approx 0.16$ to distinguish between accepting or rejecting $H_0$. In keeping with the idea of sticking with $H_0$ in the absence of strong evidence against it (low *p* values), it could be sensible to let e.g. the 90 or 95 percentile of the $\chi ^2$-distribution be the watershed between supporting $H_0$ or $H_1$. However, we think this type of argument is most reasonable for arguing for convenience null hypotheses, e.g. as a license to avoid modeling and reporting interactions if they are deemed inconsequential, and not important for the study. In this case when it is the central hypothesis we are discussing, it would seem like tilting the scales in the direction of a desired result.

The prior $p(\theta _1)$ employed in our formula is supposed to represent pre-data prior knowledge pertinent to the study. A priori we know more or less the distribution of the interest parameter for the infectious mononucleosis outcome. But we don’t know it for the interest parameter regarding the multiple sclerosis outcome (4442 cases). In the study cohort we found 103 cases of multiple sclerosis following infectious mononucleosis at age 12+ years, yielding a standardized incidence ratio of 2.35. This elevated incidence is one of the key inspirations for our hypothesis: the hypothesized protection from having siblings is supposed in a way to explain the elevated standardized incidence ratio in people having had infectious mononucleosis as a marker of delayed primary EBV infection. So according to this view a sensible value on a grid *G* on the form $\lambda =\nu /\mu \le \lambda _{max}$, with $\nu \in \{1, 2, 5, 10, 20, 50, \ldots \}$ and $\mu$ being counts of some information carrying unit in a reasonable prior and the data, respectively, would be $\lambda =100/4442=0.023$. Using this $\lambda$ yields $BF_{10}=5.65\times 10^{-5}$ and hence a vanishing probability of the alternative hypothesis. However, there are many more studies available on risk of multiple sclerosis following infectious mononucleosis, yielding remarkably similar results^34^. Taking these into account would quickly increase $\nu$ to a degree where the resulting $\lambda$ would be the same as when using the AIC. We also note that the way an inconspicuous $\chi ^2$ in this case was turned into overwhelming evidence in favor of $H_0$ exemplifies Lindley’s paradox^13^.

R code for this example is provided in Supplementary Methods.

## Discussion

The traditional frequentist hypothesis test works by collecting evidence against the null (“model criticism”); the methodology of rejecting the null hypothesis when the *p* value becomes small is the statistical equivalent of Popper’s paradigm of falsifying hypotheses. The Bayesian learning process collects evidence in favor of hypotheses; it is symmetric in the models. The frequentist approach, on the other hand, is designed to prefer a null model (for simplicity), and only to make us grudgingly be persuaded in favor of an (unspecified) alternative when the evidence is very much against the null. Our proposals are primarily intended to enhance the traditional frequentist methodology in a way that only causes us to abandon the null hypothesis if we have a specific alternative that performs noticeably better in terms of predicting the data at hand.

Bayesians have always told frequentists that it is logically wrong and unsound just to consider inferences based on the null model^24^. Viewed in that context it is slightly embarrasing to end up with an approximate expression for Bayes factor that only depends on the dimension and *p* value of the hypothesis. Having swallowed this embarrassment it is however very comforting to be able to translate or calibrate the objective *p* value for any given hypothesis to a Bayes factor and thus achieve a more realistic picture of the evidence conveyed by the data in favor of the null and the alternative hypothesis, respectively. It is actually a Bayesian solution to the Fisherian project of making statistical inference using only likelihood functions^35^!

Having constrained our Bayes factor to be monotonely increasing in the likelihood-ratio has ensured that the formula for Bayes factor for $d>1$ is the natural generalization from the case with $d=1$ where monotonicity is always fulfilled. It has also made our Bayes factor both objective, meaningful and at least corresponding to both frequentist likelihood-ratio-based and pure likelihood inference, and thus likely to be accepted by the scientific community^3,4,36^. Furthermore, this Bayes factor is easily calculated from standard statistical output, e.g. an uncategorized *p* value and dimension of the hypothesis, which is usually available in epidemiological papers, and certainly in statistical software.

**Figure 2:**
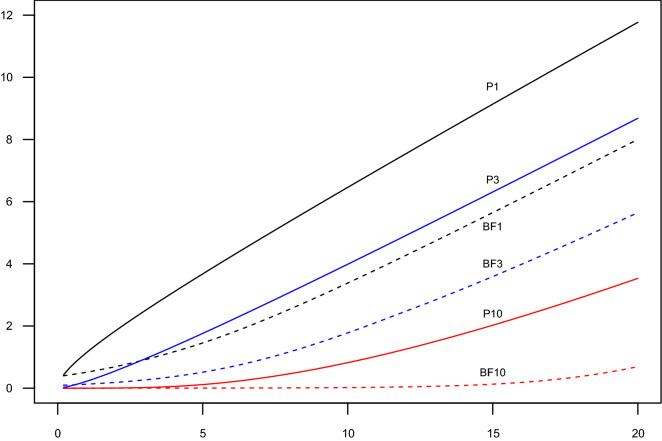
Lack of support for $H_0$ (measured as $-\log (p)$ (Pd) and $-\log (\textrm{Pr}(H_0|D))$ (BFd), respectively, as a function of $\chi ^2$ and dimension *d* of $H_1$.

How is the Bayesian inference proposed here quantitatively different from the classical frequentist inference? If we divide the parameter space into regions where we either reject or accept $H_0$ it is clear from the formula $BF_{10}=\psi ^{d/2}LR^{1-\psi }$ that the “accept” regions for the Bayesian and frequentist approaches would be of the same shape and orientation (asymptotically an ellipsoid centered at $\varvec{0}$), but with different boundaries so that the regions where we accept $H_0$ would tend to be larger in the Bayesian approach. For example, if we use Eq. (8) with $\lambda _{max}=0.255$ and $\textrm{pr}(H_1)/ \textrm{pr}(H_0)=1$ as our default methodology $BF_{10}=1$ would correspond to *p* values of 0.1573 and 0.0293 under 1- and 10-dimensional hypothesis, respectively. And $BF_{10}=19$ corresponding to $\textrm{pr}(M_0|\varvec{D})=0.05$ would correspond to *p* values of 0.0026 and $1.66\times 10^{-7}$ under 1- and 10-dimensional hypothesis, respectively. These differences in quantitative behavior between our Bayesian proposal and *p* value based methodology is illustrated in Fig. 2. If the *p* value is either very large or very small we would of course reach qualitatively the same conclusion irrespective of the chosen method. Thus if we use Bayes factor merely to choose the preferred/most likely model then the standard inference is exactly the same as when using the AIC. If we instead use Occam’s razor and only deviate from $H_0$ if there is strong evidence against it, then the Bayes factor would lead to fewer rejections of $H_0$ than when using significance testing ($p\le \alpha$) vs ($\textrm{Pr}(H_0|\varvec{D})\le \alpha$). And the evidence for the null and the alternative model is quantified in a meaningful way as probabilities; something that the traditional frequentist inference never came close to.

The mapping $(p,d)\rightarrow BF_{10}$ is monotone in *p* for fixed *d*, but is otherwise non-trivial, and therefore cannot and should not be attempted without calculating it. It would be a grave mistake and missing the point to just go on using *p* values in the belief that due to monotonicity it would lead to the same statistical inferences as using our Bayes factor.

There has been many attempts to unseat *p* values as the main vehicle for statistical inference besides confidence intervals^24,37^. This is yet another attempt to do that, and based on history is likely to fail. If it fails again it will only be because many researchers actually love all these statistically significant false positive findings in the quest for funding, promotion and what not or perhaps just lazy inertia. On the other hand it would be quite simple for journal editors and other stakeholders to recommend or require “the Bayesian version” of statistical inference presented or taken as starting point whenever a test or model choice would be deemed relevant. And maybe this methodology could also stop authors from sprinkling their texts with the (usually superfluous) words “statistically significant” when they are in fact only estimating quantities, and there is no strong evidence for some hypothesis in need of being communicated.

## Supplementary Information

Supplementary Information.

## Data Availability

All data generated or analysed during this study are included in this published article and its supplementary information files.
