# Supplementary material for: Simple nested Bayesian hypothesis testing for meta-analysis, Cox, Poisson and logistic regression models
Source: Sci Rep. 2023 Mar 23;13:4731. doi: 10.1038/s41598-023-31838-8 (PMC10036629; doi:10.1038/s41598-023-31838-8)
Supplement: Supplementary file 1 — Supplementary Information. [file 41598_2023_31838_MOESM1_ESM.pdf]

Supplementary material

for

Simple nested Bayesian hypothesis testing for meta-analysis, Cox, Poisson and logistic regression models

Klaus Rostgaard[1,2,*]

[1]Danish Cancer Society Research Center, Copenhagen, Denmark
[2]Department of Epidemiology Research, Statens Serum Institut, Copenhagen, Denmark
[*]klar@cancer.dk

ABSTRACT

Supplementary Equations E1 & E2 provides a univariate warm-up to Supplementary Equations E3-E5. The latter first provides the general closed-form expression for Bayes factor when both data and prior are assumed multivariate Gaussian. It then provides the much simpler expressions that obtains when the covariance matrices of the data and the prior are proportional. Supplementary Equation E6 examines the constraints on the prior covariance matrix $\boldsymbol{P}$ induced by a requirement that Bayes factor be monotone in the p-value. Supplementary equation E7 provides heuristic arguments why a universal Bayes factor should look like $\psi^{d/2}LR^{1-\psi}$. Finally, Supplementary Methods provides R code for the example.

Supplementary Equations E1 & E2
Expressions for Bayes factor with one-dimensional interest parameter θ

$$BF_{10} = LR(2\pi)^{-1/2}W^{-1/2}\int \exp(-\frac{1}{2}Q(\theta))d\theta$$

Let $V^{-1} \equiv K$, $W^{-1} \equiv P$, $S \equiv K + P$ and $m = S^{-1}K\hat{\theta} + S^{-1}P\theta_1$, $LR = \exp(\frac{1}{2}\theta K\theta)$. Then

$$\begin{aligned}
Q(\theta) &= (\theta-\hat{\theta})K(\theta-\hat{\theta}) + (\theta-\theta_1)P(\theta-\theta_1) \\
&= \theta S\theta - 2\theta K\hat{\theta} - 2\theta P\theta_1 + \hat{\theta}K\hat{\theta} + \theta_1 P\theta_1 \\
&= \theta S\theta - 2\theta S(S^{-1}K\hat{\theta} + S^{-1}P\theta_1) + \hat{\theta}K\hat{\theta} + \theta_1 P\theta_1 \\
&= \theta S\theta - 2\theta Sm + mSm - mSm + \hat{\theta}K\hat{\theta} + \theta_1 P\theta_1 \\
BF_{10} &= LR|W|^{-1/2}|S|^{-1/2}\exp(-\frac{1}{2}[\hat{\theta}K\hat{\theta} + \theta_1 P\theta_1 - mSm]) \\
&= |WS|^{-1/2}\exp(-\frac{1}{2}[\theta_1 P\theta_1 - mSm]) \qquad \text{(E1)}
\end{aligned}$$

Suppose further that $\theta_1 = 0$ and $P = \lambda K$ and let $\psi \equiv \lambda/(1+\lambda)$. Then

$$\begin{aligned}
BF_{10} &= |WS|^{-1/2}\exp(-\frac{1}{2}\theta_1 P\theta_1 - mSm]) \\
&= |\lambda^{-1}V(1+\lambda)K|^{-1/2}\exp(\frac{1}{2}[mSm]) \\
&= \psi^{1/2}\exp(\frac{1}{2}[mSm]) \\
&= \psi^{1/2}\exp(\frac{1}{2}[\hat{\theta}KS^{-1}K\hat{\theta}]) \\
&= \psi^{1/2}\exp(\frac{1}{2}[\frac{1}{1+\lambda}[\hat{\theta}K\hat{\theta}]) \\
&= LR\psi^{1/2}\exp(-\frac{1}{2}[\psi\hat{\theta}K\hat{\theta}]) \qquad \text{(E2)}
\end{aligned}$$

Supplementary Equations E3-E5
Expressions for Bayes factor

$$BF_{10} = LR(2\pi)^{-d/2}|\boldsymbol{W}|^{-1/2}\int \exp(-\frac{1}{2}Q(\boldsymbol{\theta}))d\boldsymbol{\theta}$$

Let $\boldsymbol{V}^{-1} \equiv \boldsymbol{K}$, $\boldsymbol{W}^{-1} \equiv \boldsymbol{P}$, $\boldsymbol{S} \equiv \boldsymbol{K}+\boldsymbol{P}$, $\boldsymbol{m} = \boldsymbol{S}^{-1}\boldsymbol{K}\hat{\boldsymbol{\theta}} + \boldsymbol{S}^{-1}\boldsymbol{P}\boldsymbol{\theta}_1$, $LR = \exp(\frac{1}{2}\hat{\boldsymbol{\theta}}^t\boldsymbol{K}\hat{\boldsymbol{\theta}})$ and let $|\cdot|$ designate determinant. Then

$$\begin{aligned}
Q(\boldsymbol{\theta}) &= (\boldsymbol{\theta}-\hat{\boldsymbol{\theta}})^t\boldsymbol{K}(\boldsymbol{\theta}-\hat{\boldsymbol{\theta}}) + (\boldsymbol{\theta}-\boldsymbol{\theta}_1)^t\boldsymbol{P}(\boldsymbol{\theta}-\boldsymbol{\theta}_1) \\
&= \boldsymbol{\theta}^t\boldsymbol{S}\boldsymbol{\theta} - 2\boldsymbol{\theta}^t\boldsymbol{K}\hat{\boldsymbol{\theta}} - 2\boldsymbol{\theta}^t\boldsymbol{P}\boldsymbol{\theta}_1 + \hat{\boldsymbol{\theta}}^t\boldsymbol{K}\hat{\boldsymbol{\theta}} + \boldsymbol{\theta}_1^t\boldsymbol{P}\boldsymbol{\theta}_1 \\
&= \boldsymbol{\theta}^t\boldsymbol{S}\boldsymbol{\theta} - 2\boldsymbol{\theta}^t\boldsymbol{S}(\boldsymbol{S}^{-1}\boldsymbol{K}\hat{\boldsymbol{\theta}} + \boldsymbol{S}^{-1}\boldsymbol{P}\boldsymbol{\theta}_1) + \hat{\boldsymbol{\theta}}^t\boldsymbol{K}\hat{\boldsymbol{\theta}} + \boldsymbol{\theta}_1^t\boldsymbol{P}\boldsymbol{\theta}_1 \\
&= \boldsymbol{\theta}^t\boldsymbol{S}\boldsymbol{\theta} - 2\boldsymbol{\theta}^t\boldsymbol{S}\boldsymbol{m} + \boldsymbol{m}^t\boldsymbol{S}\boldsymbol{m} - \boldsymbol{m}^t\boldsymbol{S}\boldsymbol{m} + \hat{\boldsymbol{\theta}}^t\boldsymbol{K}\hat{\boldsymbol{\theta}} + \boldsymbol{\theta}_1^t\boldsymbol{P}\boldsymbol{\theta}_1 \\
BF_{10} &= LR|\boldsymbol{W}|^{-1/2}|\boldsymbol{S}|^{-1/2}\exp(-\frac{1}{2}[\hat{\boldsymbol{\theta}}^t\boldsymbol{K}\hat{\boldsymbol{\theta}} + \boldsymbol{\theta}_1^t\boldsymbol{P}\boldsymbol{\theta}_1 - \boldsymbol{m}^t\boldsymbol{S}\boldsymbol{m}]) \\
&= |\boldsymbol{W}\boldsymbol{S}|^{-1/2}\exp(-\frac{1}{2}[\boldsymbol{\theta}_1^t\boldsymbol{P}\boldsymbol{\theta}_1 - \boldsymbol{m}^t\boldsymbol{S}\boldsymbol{m}]) \qquad \text{(E3)}
\end{aligned}$$

Suppose further that $\boldsymbol{P} = \lambda\boldsymbol{K}$ and hence $\boldsymbol{S} = (1+\lambda)\boldsymbol{K}$ and thus $\boldsymbol{m} = \frac{1}{1+\lambda}\hat{\boldsymbol{\theta}} + \frac{\lambda}{1+\lambda}\boldsymbol{\theta}_1$ and let $\psi \equiv \lambda/(1+\lambda)$. Then

$$\begin{aligned}
BF_{10} &= |\lambda^{-1}\boldsymbol{V}(1+\lambda)\boldsymbol{K})|^{-1/2}\exp(-\frac{1}{2}[\boldsymbol{\theta}_1^t(\lambda\boldsymbol{K})\boldsymbol{\theta}_1 - \boldsymbol{m}^t((1+\lambda)\boldsymbol{K})\boldsymbol{m}]) \\
&= \psi^{d/2}\exp(\frac{1}{2}[\lambda\boldsymbol{\theta}_1^t\boldsymbol{K}\boldsymbol{\theta}_1 - (1+\lambda)\boldsymbol{m}^t\boldsymbol{K}\boldsymbol{m}]) \qquad \text{(E4)}
\end{aligned}$$

Suppose now further that $\boldsymbol{\theta}_1 = \boldsymbol{0}$. Then we obtain

$$\begin{aligned}
BF_{10} &= \psi^{d/2}\exp(\frac{1}{2}[\frac{1}{1+\lambda}\hat{\boldsymbol{\theta}}^t\boldsymbol{K}\hat{\boldsymbol{\theta}}]) \\
&= LR\psi^{d/2}\exp(-\frac{1}{2}[\psi\hat{\boldsymbol{\theta}}^t\boldsymbol{K}\hat{\boldsymbol{\theta}}]) \qquad \text{(E5)}
\end{aligned}$$

Supplementary Equation E6
Constraints on P when Bayes factor shall be monotone in p

Let $\boldsymbol{\theta} = t\boldsymbol{u}$, $\boldsymbol{u}$ a unit vector, $t \geq 0$. Use the basis where $\boldsymbol{K}$ is represented as $\boldsymbol{\Lambda} = \text{diag}(\lambda_1,\ldots,\lambda_d)$ with $\lambda_1 \geq \ldots \geq \lambda_d$. Consider any two-dimensional margin in this basis of $\boldsymbol{K}+\boldsymbol{P}$ with indices a and b, $a < b$: Then $\boldsymbol{u}^t = (u_a, u_b) = (x, \pm\sqrt{1-x^2})$ and

$$(\boldsymbol{O}^t(\boldsymbol{K}+\boldsymbol{P})\boldsymbol{O})_{ab} = \begin{pmatrix} \lambda_a & 0 \\ 0 & \lambda_b \end{pmatrix} + \begin{pmatrix} \psi_{aa} & \psi_{ab} \\ \psi_{ab} & \psi_{bb} \end{pmatrix} = \boldsymbol{Q}_{ab}$$

We want to have $\boldsymbol{K}+\boldsymbol{P}$ obey the constraint that $\boldsymbol{u}^t\boldsymbol{Q}_{ab}\boldsymbol{u}$ is monotonically decreasing in p and hence in $\boldsymbol{u}^t(\boldsymbol{O}^t\boldsymbol{K}\boldsymbol{O})_{ab}\boldsymbol{u} = \lambda_a x^2 + \lambda_b(1-x^2)$ which is monotonically decreasing from $x=-1$ to $x=0$ and monotonically increasing from $x=0$ to $x=1$,

and constant throughout when $\lambda_a = \lambda_b$ in which case p is also constant. The constraint implies that $[\boldsymbol{u}^t(\boldsymbol{O}^t(\boldsymbol{K}+\boldsymbol{P})\boldsymbol{O})_{ab}\boldsymbol{u}](x)$ should be decreasing between $x=-1$ and $x=0$ and increasing between $x=0$ and $x=1$.

$$\begin{aligned}
[\boldsymbol{u}^t(\boldsymbol{O}^t(\boldsymbol{K}+\boldsymbol{P})\boldsymbol{O})_{ab}u](x) &= \lambda_a x^2 + \lambda_b(1-x^2) + \psi_{aa}x^2 \\
&\quad + \psi_{bb}(1-x^2) + 2\psi_{ab}x\sqrt{1-x^2} \\
\frac{\partial}{\partial x}\boldsymbol{u}^t\boldsymbol{Q}_{ab}\boldsymbol{u} &= 2(\lambda_a + \psi_{aa} - \lambda_b - \psi_{bb})x \\
&\quad + 2\psi_{ab}[\sqrt{1-x^2} - x/\sqrt{1-x^2}] \qquad \text{(E6)}
\end{aligned}$$

which only has a root in $x=0$ as required, when $\psi_{ab}=0$. Repeating the argument for all two-dimensional margins shows that the constraint implies that 1) $\boldsymbol{P}$ is diagonal in the same basis as $\boldsymbol{K}$, 2) whenever $\lambda_a = \lambda_b$ then $\psi_{aa} = \psi_{bb}$ and 3) $\lambda_a + \psi_{aa} > \lambda_b + \psi_{bb}$ whenever $\lambda_a > \lambda_b$. It is easily seen that for 3) to hold for any scale copy of $\boldsymbol{P}$ we must have $\psi_{11} \geq \psi_{22} \geq \ldots \geq \psi_{pp}$.

Supplementary Equation E7
Further heuristic arguments why a universal Bayes factor should be $\psi^{d/2}LR^{1-\psi}$

Consider a unimodal log-likelihood function $\ell(\cdot)$ maximized by $\boldsymbol{\theta} = \hat{\boldsymbol{\theta}}$ with Hessian $-\boldsymbol{K}$ in $\boldsymbol{\theta} = \hat{\boldsymbol{\theta}}$ and choose accordingly a prior $p(\boldsymbol{\theta})$ on the form $N_d(\boldsymbol{0}, \lambda^{-1}\boldsymbol{K}^{-1})$ and assume the posterior $p(\boldsymbol{\theta}|\boldsymbol{D})$ unimodal too with maximizer $\bar{\boldsymbol{\theta}}$. Assume no nuisance parameters. Let LR denote the constant $L(\hat{\boldsymbol{\theta}})/L(\boldsymbol{0})$ and $LR(\boldsymbol{\theta})$ the function $L(\boldsymbol{\theta})/L(\hat{\boldsymbol{\theta}})$. According to the Savage-Dickey density ratio theorem we have

$$\begin{aligned}
BF_{10} &= p(\boldsymbol{0})/p(\boldsymbol{0}|\boldsymbol{D}) = p(\boldsymbol{0})/(\frac{LR(\boldsymbol{0})p(\boldsymbol{0})}{\int LR(\boldsymbol{\theta})p(\boldsymbol{\theta})d\boldsymbol{\theta}}) = LR\int LR(\boldsymbol{\theta})p(\boldsymbol{\theta})d\boldsymbol{\theta} \\
&= LR(2\pi\lambda)^{d/2}|\boldsymbol{K}|^{1/2}\int LR(\boldsymbol{\theta})\exp(-\frac{\lambda}{2}\boldsymbol{\theta}^t\boldsymbol{K}\boldsymbol{\theta})d\boldsymbol{\theta} \\
&= LR(\frac{\lambda}{1+\lambda+b_1})^{d/2}LR(\bar{\boldsymbol{\theta}})\exp(-\frac{\lambda}{2}\bar{\boldsymbol{\theta}}^t\boldsymbol{K}\bar{\boldsymbol{\theta}}) = (\frac{\lambda}{1+\lambda+b_1})^{d/2}LR^{1-\frac{\lambda}{1+\lambda}+b_2} \qquad \text{(E7)}
\end{aligned}$$

where b_1 is defined by the above expression and tends to 0 as the Laplace approximation of the integral improves. And the corrective of LR: $-\frac{\lambda}{1+\lambda}+b_2 < 0$ because both $LR(\bar{\boldsymbol{\theta}})$ and $\exp(-\frac{\lambda}{2}\bar{\boldsymbol{\theta}}^t\boldsymbol{K}\bar{\boldsymbol{\theta}})$ are less than 1. Furthermore this corrective tends to 0 as $\lambda \downarrow 0$ because both terms tend to 1 then. The semblance of this expression to our proposed universal Bayes factor (where $b_1 = b_2 = 0$) is obvious.

Supplementary Methods
R code for the example

Future versions of the three R functions below are intended to be included in the EpiForsk R package to appear. From experience we know that Bayes factors can be astronomically large. We therefore prefer always to first calculate the logarithm of Bayes factor.

```
logasympBF=function(chisq,d=1,lambda=NA,lambdamax=0.255){
  chisq<-abs(chisq)
  lambda<-abs(lambda)
  lambdamax<-abs(lambdamax)
  if (is.na(lambda)) {lambda<-d/max(chisq,10E-6)}
  lambda0<-min(lambda,lambdamax)
  psi<-lambda0/(1+lambda0)
logasympBF<-d/2*log(psi)+(1-psi)*chisq/2
return(logasympBF)}

asympBF=function(chisq,d=1,lambda=NA,lambdamax=0.255){
  asympBF<-exp(logasympBF(chisq=chisq,d=d,lambda=lambda,lambdamax=lambdamax))
  return(asympBF)}
```

```
watershed=function(chisq){
  psi<-0.5
  delta<-0.5
  twologBF<-log(psi)+chisq*(1-psi)
  taeller<-1;
  while((abs(twologBF)>10e-6)*(taeller<20))
  {
    taeller<-taeller+1
    delta<-delta/2;
    if (twologBF<0) {psi<-psi+delta} else {psi<-psi-delta}
    twologBF<-log(psi)+chisq*(1-psi)
  }
  lambda<-psi/(1-psi)
  return(lambda)
}

# main example
d<-8
chisq<-qchisq(1-0.19,8)
chisq
lambda<-min(d/chisq,0.255)
lambda
psi<-lambda/(lambda+1)
psi
logbf<-logasympBF(chisq=chisq,d=8,lambda=lambda)
logbf
bf<-exp(logbf)
bf
probH1<-bf/(1+bf)
probH1
probH0<-1-probH1
probH0

# lambda not constrained by AIC - lambdamax=100
lambda<-d/chisq
lambda
psi<-lambda/(lambda+1)
psi
logbf<-logasympBF(chisq=chisq,d=8,lambda=lambda,lambdamax=100)
logbf
bf<-exp(logbf)
bf
probH1<-bf/(1+bf)
probH1
probH0<-1-probH1
probH0

# trying to construct a practically null result
# from Rostgaard 2022 0.2 y younger siblings
deltalogHR<--0.2*log(0.80)
sigma<-(log(1.19)-log(0.89))/3.92
chisq=(deltalogHR/sigma)**2
log(0.80)
deltalogHR
sigma
```

```
chisq
1/chisq
watershed(chisq)
# leads nowhere useful chisq=0.36

# lambda on a grid
lambda<-100/4442
lambda
chisq<-qchisq(1-0.19,8)
bf<-asympBF(chisq=chisq,d=8,lambda=lambda)
bf
```
